# Pancreatic Adeno-MiNEN, a Rare Newly Defined Entity with Challenging Diagnosis and Treatment: A Case Report with Systematic Literature Review and Pooled Analysis

**DOI:** 10.3390/jcm11175021

**Published:** 2022-08-26

**Authors:** Roberta Angelico, Leandro Siragusa, Cristine Brooke Pathirannehalage Don, Bruno Sensi, Federica Billeci, Leonardo Vattermoli, Belen Padial, Giampiero Palmieri, Alessandro Anselmo, Alessandro Coppola, Giuseppe Tisone, Tommaso Maria Manzia

**Affiliations:** 1 HPB and Transplant Unit, Department of Surgical Sciences, University of Rome Tor Vergata, 00133 Rome, Italy; 2 Department of Diagnostic Imaging and Interventional Radiology, Tor Vergata University of Rome, 00133 Rome, Italy; 3 Histopathologic Unit, Tor Vergata University of Rome, 00133 Rome, Italy; 4 Department of Surgery, University Campus Bio-Medico of Rome, 00128 Rome, Italy

**Keywords:** pancreatic tumour, mixed neuroendocrine-non neuroendocrine neoplasm, MiNEN

## Abstract

Mixed neuroendocrine non-neuroendocrine neoplasms (MiNEN) are a peculiar entity that can occur throughout the whole gastrointestinal trait, and pancreatic localization is rare. Their main characteristic is the presence of at least a neuroendocrine and an epithelial component, each accounting for at least 30% of the tumour mass. The presence of epithelial ductal component defines adeno-MiNEN. We report a case of a 59-year-old woman affected by pancreatic adeno-MiNEN with challenging diagnosis and successfully treated. A systematic literature review and pooled analysis was also performed, aiming to define the management and outcomes of pancreatic adeno-MiNEN. Out of 190 identified records, 15 studies including 28 patients affected by pancreatic-adeno-MiNEN were included in the analysis. Pancreatic adeno-MiNEN occurred mainly in males (82.8%) and at a mean age of 61.7 (range: 24–82) years. Pre-operative diagnosis was possible only in 14.2% of cases. At presentation, the majority had already advanced disease (TNM stage III (53.8%) and stage IV 19.3%). Adjuvant therapy was performed in 55% of patients, and the tumour recurrence rate was in 30% of cases. Median disease-free survival (DFS) was 12 months (range: 0–216 months) with a 5-year DFS of 16.6%, while the median overall survival (OS) was 12 months (range: 0–288 months) with a 5-year OS of 23.5%. Pancreatic adeno-MiNENs are rare; as they have very heterogenous behaviour, they are rarely diagnosed preoperatively and have poor prognosis. Treatment of localised MiNEN still relies on radical surgical resection, which seems essential to achieve a good oncological prognosis. International registry on MiNEN is necessary to improve the knowledge on this rare tumour and to improve its outcomes.

## 1. Introduction

Mixed neuroendocrine-non-neuroendocrine neoplasms (MiNEN) are a rare subtype of neuroendocrine neoplasms that may occur in any part of gastrointestinal tract and have great heterogeneity. The histological features of MiNEN were newly defined in 2017 by the 4th World Health Organization (WHO) classification for tumours of endocrine organs from the old definition of mixed adeno-neuroendocrine carcinoma (MANEC) [1]. Its main characteristic is the presence of at least two neoplastic components, a neuroendocrine and an epithelial one, each accounting for at least 30% of the tumour mass [2]. The epithelial component could comprise an acinar neoplasia (acinar-MiNEN), which is the most common, or a ductal one (adeno-MiNEN). Usually, MiNENs have a rapidly progressive behaviour needing aggressive treatment; however, data are still relatively lacking so that natural history still remains unclear. Preoperative diagnosis of MiNEN is very rare, and its treatment is mostly based on post-operative pathological examination. Similarly, the literature about pancreatic MiNEN is rare, and only a limited number of cases is reported.

We present a rare case of a 59-year-old woman affected by a locally advanced pancreatic adeno-MINEN. A systematic review of the literature and pooled analysis on pancreatic adeno-MiNEN is also reported, aiming to define the management and outcome of this rare neoplasm.

## 2. Case Report

### 2.1. Clinical Presentation

A 59-year-old woman was referred to our institution for jaundice and dark urine output in the previous two months. The patient had no relevant medical and surgical history. At physical examination, she was frankly jaundiced with a palpable gallbladder. Blood tests revealed elevated bilirubin (14.57 mg/dL) and liver function tests (aspartate transaminase: 138 U/L; alanine transaminase: 112 U/L). Screening of tumoral markers showed an elevated cancer antigen 19-9 (CA19-9) (171 U/mL (normal range: <35.0 U/mL)) and carcinoembryonic antigen (CEA) (7.23 ng/mL (normal range: <3.00 ng/mL)), but chromogranin A (CgA) was within normal range (20 ng/mL (normal range: 11.80–88.00 ng/mL)).

**Figure 1:**
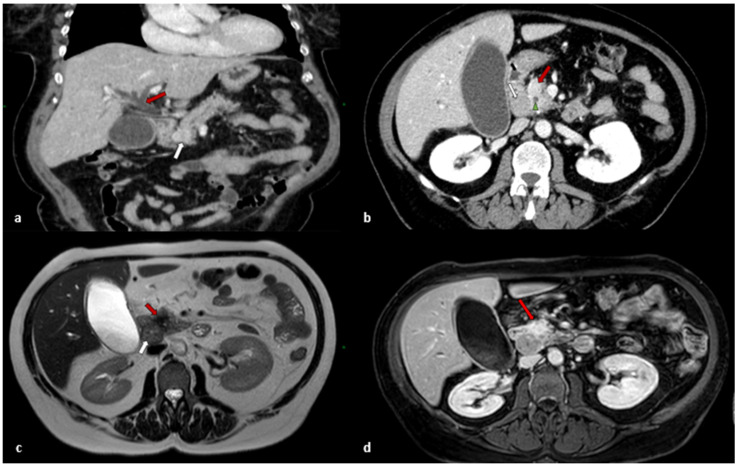
(**a**) Post-contrastographic CT scan coronal view shows inhomogeneous enhanced expansive solid mass with irregular edges localized at pancreatic cephalic portion (white arrow). The mass exerts compressive effect on the duodenum and choledochus, with evident dilatation of extra- and intra-hepatic biliary ducts upstream (red arrow); (**b**) Contrast-enhanced CT scan axial view shows inhomogeneous enhanced mass with irregular edges localized at cephalic portion of pancreas (red arrow). Made evident are the dilated choledochus in the context of the mass (green arrowhead) and the presence of enlarged, globose, and inhomogeneous lymph node (white arrow) localized in peri-duodenal region, which is suspicious for metastasis; (**c**) MRI axial T2-weighted image shows the lesion as hypointense area with badly defined spiked edges lesions (red arrow). It also shows the enlarged, globose metastatic lymph node, which appears slightly and inhomogeneously hypointense (white arrow); (**d**) MRI gadolinium-enhanced T1-weighted axial image with fat suppression shows intense signal enhancement of the lesion (red arrow).

A contrast-enhanced computer tomography (CT) scan was performed and reported a 2.5 cm irregular hyperdense mass located in the head of the pancreas with upstream intra-hepatic biliary tract and common bile duct (14 mm) (CBD) dilation. No further lesions were found except for a 1 cm mass close to the superior mesenteric vein with conserved cleavage plane, which was identified as a node (Figure 1a,b). A liver magnetic resonance imaging (MRI) was then performed, confirming the presence of a 20 mm pancreatic head mass with a suspicious node next to the uncinate process and inferior cava vein (IVC) and dilatation of Wirsung’s duct (Figure 1c,d).

Endoscopic ultrasound (EUS) was performed showing a 15 × 15 mm irregular hypoechoic area within the pancreatic parenchyma that infiltrated the distal common bile duct (CBD) and that appeared in continuity with an irregular hypoechoic mass (28 × 22 mm) in the peri-duodenal area, next to the pancreatic head and IVC. The latter was suspected of being a metastatic adenopathy and was biopsied but not diagnostic.

Endoscopic retrograde cholangiopancreatography (ERCP) showed a tight distal CBD stenosis. At this time, a biopsy of the distal CBD was performed, and a 10 Fr prosthesis was placed in the CBD. An additional 5 Fr plastic prosthesis was placed in the Wirsung duct for the prevention of post-ERCP pancreatitis. Distal CBD biopsy resulted in neoplastic tissue with neuroendocrine differentiation, panCK+, synaptophysin (Syn)+, chromogranin A (CgA)+, CD56−, and Ki67 70%.

### 2.2. Treatment

After a multi-disciplinary team discussion of the case, a pancreaticoduodenectomy was planned. Intraoperatively, the pancreatic head appeared soft and the CBD visibly enlarged. There was no sign of liver metastases or peritoneal carcinosis. A pylorus-preserving R0 pancreaticoduodenectomy was performed with associated lymphadenectomy of right (n:8) and left paracardial (n:9) areas, common hepatic artery (n:12), splenic artery (n:13), and paraaortic region (n:16).

The post-operative course was uneventful. On the first post-operative day (POD), the cholestatic indicator down-trended. The hepatic–jejunal anastomosis drain and naso–jejunal tube were removed on POD 3, followed by the pancreato–gastric drainage and naso–gastric tube on POD 6. A liquid diet was started 3 days after surgery and a solid diet at POD 4. Before discharge, an abdominal X-ray confirmed the correct position of the pancreatic prosthesis. The patient was discharged on POD 8 in good clinical condition.

### 2.3. Histological Findings

**Figure 2:**
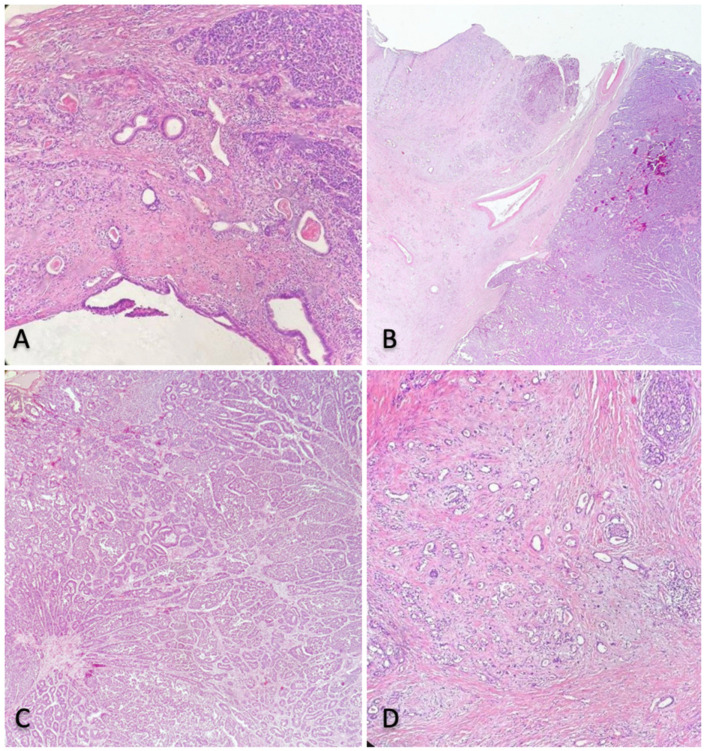
(**A**,**B**) MiNEN consisting of a neuroendocrine component (both of the left side of the images) and non-neuroendocrine component (ductal adenocarcinoma) (both on the right side of the images), each one accounting for more than 30% of the tumour. (**C**) Neuroendocrine component; (**D**) Non neuroendocrine, ductal adenocarcinoma component.

Histological examination revealed a 25 mm mixed neuroendocrine non-neuroendocrine neoplasia (MiNEN) with two different neoplastic components: neuroendocrine carcinoma (NEC) and high-grade ductal adenocarcinoma (Figure 2).

**Figure 3:**
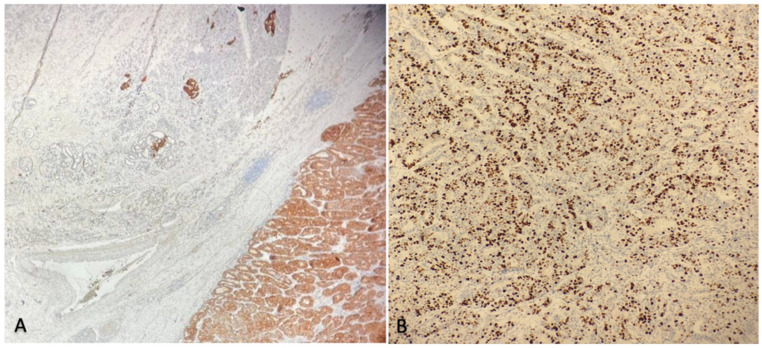
(**A**) Synaptophysin staining showing a diffuse positivity in the neuroendocrine component (on the right) in contrast to the ductal adenocarcinoma component (on the left); (**B**) Ki 67 immunostaining having a proliferation index 70%.

On immunohistochemistry, the ductal structures were positive for CK-7, CK-19, and EMA, while NEC cells were immunoreactive to CgA and Syn. The neuroendocrine carcinoma component had a 70% Ki-67 rate, with focal angio-invasion and infiltration of a peripancreatic node (Figure 3).

This component originated from peri-ductal pancreatic tissue and appeared separated from pancreatic parenchyma by a pseudo-capsule. There was no sign of duodenal infiltration, but the duodenal component in proximity of the tumour was the site of a large abscess extending up to the overlying mucosa, which was focally ulcerated. The ductal adenocarcinoma derived from main pancreatic duct and appeared to infiltrate the surrounding pancreas with perineural invasion.

Fifteen nodes were resected and analysed. There were five positive nodes, and among these, four positive nodes were found in peri-pancreatic tissues, appearing as metastases morphologically referable to the neuroendocrine component (synaptophysin+), while one positive node adjacent to the hepatic artery showed sites of metastases deriving from the adenocarcinoma. Hence, the tumour was classified as a pT2N1M0.

### 2.4. Outcomes and Follow-Up

Post-operatively, adjuvant chemotherapy with cisplatinum and etoposide was proposed to the patient, but the treatment was refused by the patient for personal reasons despite being fully informed of the potential risk of tumour recurrence. Therefore, a strict follow-up was planned. At 12-months of follow-up, PET-CT scan did not show any sign of recurrence. Twelve months after surgery, the patient is alive and in good general condition.

## 3. Materials and Methods

**Figure 4:**
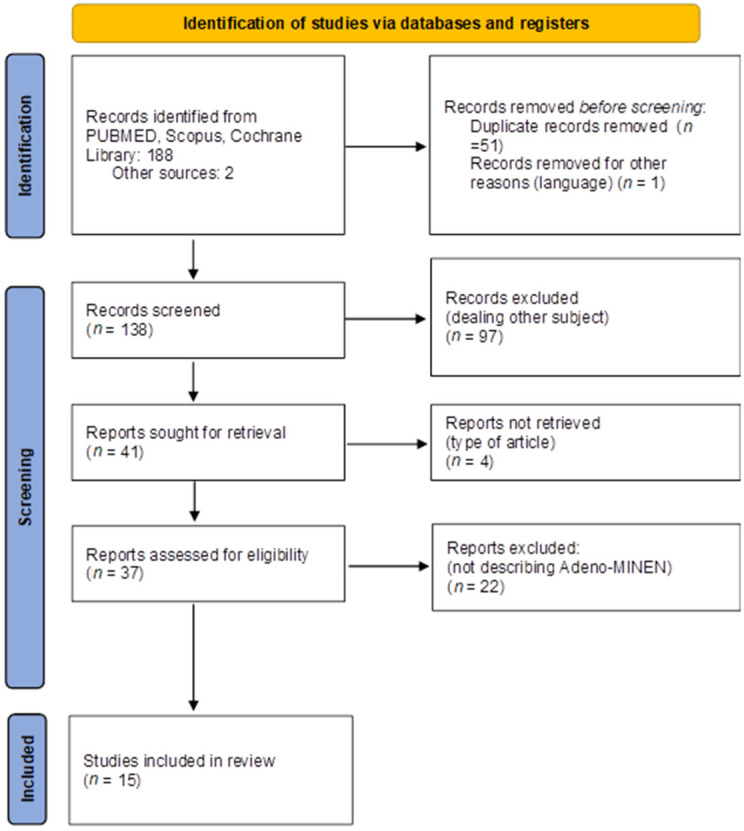
PRISMA 2020 flow diagram of systematic search.

A systematic literature review was performed in accordance with the current Preferred Reporting Items for Systematic Reviews and Meta-analyses (PRISMA 2020) guidelines for systematic reviews (Figure 4). This systematic review was registered on PROSPERO under the number of protocols CRD42022333788.

### 3.1. Search Strategy

Searches were conducted for all English-language, full-text articles published until 31 March 2022. The following database sources were searched: *PubMed* (*MEDLINE*), *Scopus*, and *Cochrane Library*.

The following term combinations were used: (Mixed neuroendocrine non-neuroendocrine tumour pancreas), (Mixed neuroendocrine non neuroendocrine pancreas), (MINEN pancreas), (Adeno MINEN pancreas), (Mixed adenocarcinoma non neuroendocrine pancreas), (Mixed ductal pancreatic carcinoma), (Mixed ductal-pancreatic tumour), (Mixed ductal-endocrine carcinoma pancreas), (Mixed exocrine-endocrine pancreas), and (Mixed adeno-neuroendocrine carcinoma). Furthermore, the references list of each selected article was analysed to identify additional relevant studies. Records were screened for relevance based on their title and abstract, and successively, the full text of the remaining articles was analysed.

### 3.2. Inclusion and Exclusion Criteria

The type of studies eligible for inclusion was all original studies (retrospective, prospective, randomised clinical trials, case series, and case report) reporting pancreatic adeno-MiNEN cases. Narrative and systematic reviews and metanalysis were excluded as well as any article in which clear features of adeno-MiNEN were not clearly defined. Two authors (C.P.D. and L.S.) independently screened each record from full-text articles for eligibility and extracted the data, including quality analysis. Disagreement was resolved by discussion and consensus; if no agreement was reached, a third author was consulted (R.A.).

### 3.3. Data Extraction and Synthesis

Each article was carefully read and analysed independently by two authors (C.P.D. and L.S.) in an effort to identify all pancreatic adeno-MINEN cases. Demographic; pre-, intra-, and post-operative data; and oncological follow up were extracted and analysed. Descriptive statistics were produced from the dataset: continuous data were pooled and are reported as percentages. There was no comparative statistical analysis.

### 3.4. Endpoint

The primary aims were to identify and analyse management and outcome of all pancreatic adeno-MiNEN cases defined per WHO classification as pancreatic neuroendocrine tumour with two neoplastic components, i.e., a neuroendocrine and a ductal one, each accounting for at least 30% of the tumour component [2].

## 4. Results

One-hundred and ninety records were identified from database search. Fifty-two records were removed before screening due to being duplicates (n = 51) or written in other language (n = 1). Among 138 records screened by abstract, 97 articles were excluded as not-pertinent. After excluding another 22 not describing adeno-MiNEN, 15 studies met eligibility criteria and were included in the analysis. Study selection is summarized in Figure 4.

Twenty-eight cases of pancreatic adeno-MiNEN have been reported (Table 1) [3,4,5,6,7,8,9,10,11,12,13,14,15,16,17].

Pancreatic adeno-MiNEN occurs mainly in males (male 82.8% vs. female 18.2%) and at a mean age of 61.7 (range: 24–82). Presentation symptoms are abdominal pain in 33.3% of cases, obstructive jaundice in 33.3% of cases, incidental diagnosis in 25% of cases, weight loss and anaemia in 16.6% of patients, respectively; and nausea and vomiting in 8.3% of cases. Pre-operative diagnosis was possible in 14.2% of cases. MiNEN location was mainly in head of pancreas (63.6%), and almost one-fifth of cases were located in the body (18.2%) and tail of pancreas (18.2%).

Regarding pTNM staging, 7.7% of patients were stage I, 19.2% were stage II, more than half were stage III (53.8%), and 19.3% were stage IV. Most of MiNENs were G3 (79.2%), while 12.5% were G2, and only 8.3% were G1. The majority of patients underwent pancreaticoduodenectomy (57.1%), and the other underwent distal splenopancreasectomy (28.7%), total pancreasectomy (7.1%), and debulking procedures (7.1%). In one case, the type of surgical resection was not specified. Neoadjuvant therapy was never performed, while 55% of patients underwent adjuvant therapy. Recurrence occurred in six cases (30%): three patients had liver recurrence, one had local recurrence with consensual peritoneal carcinosis, one had local recurrence with lung and liver metastasis, and in one case, cancer recurred, but location was not specified.

The median disease-free survival (DFS) was 12 months (range: 0–216 months): 1-year DFS was 62.5% of cases, 2-year DFS was 41.6% of cases, and 5-year DFS was 16.6% of cases. The median overall survival (OS) was 12 months (range: 0–288 months): 1-year OS was 84% of cases, 3-year OS was 27.7% of cases, and 5-year OS was 23.5% of cases. According to latest follow up reported, death occurred in 61.5% of cases.

## 5. Discussion

The first description of a gastrointestinal tumour with both neuroendocrine and exocrine component was by Cordier in 1924 [18]. Initially, these mixed tumours were defined under the term of mixed adeno-neuroendocrine carcinoma (MANEC). In 2017, the WHO Classification of Tumors of Endocrine Organs introduced the term “MiNEN” for the first time and also extended the concept of mixed neuroendocrine-non-neuroendocrine tumours to all gastrointestinal organs [2,19].

MiNEN pathogenesis is still unclear, but different theories have been proposed to allow a better understanding of the double morphology of these tumours. The “collision theory” hypotheses that MiNEN might be the consequence of the combined growth of two different tumoral cell populations; instead, the “common precursor theory” sustains that the tumour could derive from the proliferation of a single totipotent pancreatic stem cell [18].

MiNEN can be morphologically classified in three different categories: (1) collision MiNENs, where a juxtaposition exists of the two malignant cell populations without any mixing, and in this case, the populations do not derive from a common precursor; (2) composite MiNENs, where the two different components create an intermingled population or a predominant population recognizable in a focal area; and (3) amphicrine MiNENs, where there is only one population, but cells have phenotypes belonging to two different types of malignancies [2,20].

WHO classification divides neuroendocrine neoplasms (NENs) into well- and poorly differentiated according to their grading and mitotic rate [21]. Pancreatic MiNENs can be combinations between neuroendocrine neoplasms (NETs) or neuroendocrine carcinomas (NECs) with pancreas carcinomas (ductal adenocarcinoma, acinar cell carcinoma, intraductal papillary mucinous neoplasm, and serous cystic neoplasm) [18,20].

Recently, La Rosa et al. created a classification of MiNENs according to their grade of malignancies [2,20]. High-grade MiNEN typically are the most frequent, being composed by a non-neuroendocrine carcinoma or adenoma and NEC. In such cases, the NEC component is the most aggressive one, as occurred in our case where a MiNEN comprising NEC and high-grade ductal adenocarcinoma was found. Intermediate-grade MiNEN usually combines a non-neuroendocrine carcinoma with a well-differentiated NET, and in this case, the prognosis often depends on the non-neuroendocrine component, whereas G3 is a negative prognostic factor. Finally, low-grade MiNEN combines an adenoma with a well-differentiated NET [2,22].

Pancreatic MiNEN are very rare, as they represent only 0.5% of all pancreatic malignancies and 5% of all pancreatic NEN (which themselves account for 1% of all gastrointestinal malignant tumours), and among these, adeno-MiNEN are the rarer subtype [2,11].

To the best of our knowledge, 28 cases of pancreatic adeno-MiNENs have been reported so far in the literature. Our review clearly shows that MiNENs are aggressive tumours (53.8% Stage III; 79.2% G3), presenting more frequently in men (>80% of cases) of around 60 years of age. Moreover, MiNENs affect predominantly the pancreatic head region and therefore are treated mostly by pancreaticoduodenectomy procedures.

Yet, only 14% of patients undergo surgery with a pre-operative diagnosis. Imaging studies such as CT scan and MRI are important for MiNEN detection though they are not able to distinguish them from adenocarcinoma [23]. Biopsies performed by EUS-FNAB/ERCP-brushing may orienteer the diagnosis, but rarely do they lead to a definitive one. This highlights the difficulty in specific diagnosis of MiNEN without complete pathological evaluation, which is the gold-standard diagnostic modality. Tissue diagnosis require haematoxylin and eosin stains to demonstrate the neuroendocrine phenotype along with immune histochemistry for synaptophysin and chromogranin-A and for carcinoma markers [18,21]. Diagnostic criteria are challenging, necessitating both NET and carcinoma components to represent at least 30% of the cell mass, and they may be particularly difficult to fulfil when analysing a limited tissue biopsy, such as from EUS-FNAB/ERCP-brushing samples. Nonetheless a strong effort to obtain early diagnosis with EUS-FNAB and ERCP-brushing samples is of significant importance, especially in those 26% of cases presenting at an early (I/I) stage. These tumours may be misrecognized as “simple” NETs and therefore under-treated or unduly delayed. In fact, while the European Neuroendocrine Tumour Society (ENETS) 2017 guidelines recommend that pancreatic NET > 2 cm should undergo oncological resection associated with central lymphadenectomy of at least 12 nodes, smaller NET may undergo more conservative treatment, which would be inappropriate for MiNEN [23]. In fact, although surgical indications are not well-defined for MiNENs, according to De Mestier et al., high-grade and intermediate-grade localized MiNENs should be treated with curative, intensive surgery if feasible [22]. For this reason, it is also fundamental to examine the biopsy samples with a full immune-histochemical panel, including both neuro-endocrine and carcinoma markers.

In our case, pre-operative biopsy showed both neuroendocrine (Syn and CgA) and carcinoma (panCK+) markers, but the cyto/histology were predominant of NET, and there was no ground (<30% carcinoma component) for a formal MiNEN diagnosis, and we could prove a diagnosis of adeno-MiNEN only at the post-operative histological examination. This should bring attention to the fact that when both markers are present despite a presumptive NET diagnosis, and especially in the presence of high-grade lesions, a high index of suspicion for MiNEN should be kept in mind. On the other hand, when ductal adenocarcinoma is suspected, given its dismal prognosis and its radical treatment, misdiagnosis should not be a problem, as it would not result in under-treatment of the MiNEN. In fact, among published cases, curative surgery appears possible, more likely than in ductal adenocarcinoma and less so when compared to NET: median disease-free survival is 12 months, while overall survival at 5 years amounts to 23% [24,25].

Once diagnosis is achieved, it is important to rely on MiNENs’ classification because of its prognostic value in stratifying patients for therapeutic decision making. A NET-dedicated multidisciplinary group should discuss the patient, relying especially on the histopathological features of the tumour in order to evaluate its potential to metastasize. Our case also shows the importance of a radical surgical resection (R0) as well as an adequate TNM staging through nodules excision. Interestingly, we found both tumoral components in different nodules supporting the “collision theory” for the MiNENs pathogenesis.

Neoadjuvant chemotherapy has never been performed so far for pancreatic adeno-MiNENs (even when pre-operative diagnosis is obtained); therefore, it should only be taken into consideration in the setting of a clinical trial. Adjuvant chemotherapy is used in 55% of cases, but chemotherapy regimens are non-standardised, and different strategies are reported. It is still unclear whether therapy should target both components of MiNENs or the major/worse component only. La Rosa et al. proposed that chemotherapy should focus on the dominant neoplastic component because the outcomes of the tumour tend to follow the behaviour of the latter [18]. Many studies support that a combination of systemic, platinum-based chemotherapy associated with local treatment (radiotherapy, surgery, or both) can offer the best chance of long-term survival [26,27].

Unfortunately, patients undergo non-radical resection (including biopsy and debulking) in 7–14% of cases. Up-front systemic chemotherapy is reserved for advanced disease. Usually, for high-grade MiNENs with a predominant NEC component, first-line chemotherapy should combine etoposide to cisplatine or carboplatine; when the predominant component is a non-neuroendocrine carcinoma, chemotherapy should be similar to that proposed to “pure” carcinoma of the same localization. Intermediate-grade MiNENs undergo systemic chemotherapy that targets the components identified in metastases or to both constituents of the tumour [22].

Overall, evidence guiding diagnosis and management of pancreatic MiNENs is still scarce, and the establishment of national and international registries is mandatory to usher progress in this evolving oncological field.

## 6. Conclusions

Pancreatic adeno-MiNENs are a rare subtype of neuroendocrine neoplasms having a very heterogenous behaviour and are mainly aggressive with poor prognosis. Preoperative diagnosis is infrequent, and its treatment relies mainly on radical surgical resection, which seems essential to achieve a good oncological prognosis. However, the number of cases reported is still too low to draw any significant conclusion, so national and international registry are necessary to improve MiNENs’ knowledge: shared data may highlight the most appropriate management options, help achieving a standardized approach to these tumours, and ultimately lead to improved outcomes.

## Figures and Tables

**Table 1:** Literature review of pancreatic adeno-MiNEN.

Authors and Year	Type of Study	N of Pts	Sex	Age (Years)	Presentation	Preoperative Diagnosis	TNM-Staging	G	Pancreatic Localization	Surgical Treatment	Adjuvant Treatment	Tumour Recurrence	Follow-Up	Death
Terada et al., 1999 [3]	Case report	1	M	24	Anaemia	No	nr	nr	Head	PD	nr	Yes, local, lung, liver	DFS 216 months OS 288 months	Yes
Chatelain et al., 2002 [4]	Case report	1	F	76	Incidental finding	No	T3N0M0 Stage II	nr	Tail	DPS	No	nr	nr	nr
Terada et al., 2002 [5]	Case report	1	M	34	Abdominal pain	Yes	T1N0M0 Stage I	G2	Body	DPS	No	nr	nr	nr
Ballas et al., 2005 [6]	Case report	1	F	65	Abdominal pain, anaemia, nausea	No	T4N0M0 Stage III	nr	Tail	DPS	nr	No	DFS 18 months OS 18 months	No
Hashimoto et al., 2008 [7]	Case report	1	M	75	Obstructive jaundice	No	T3N1M0 Stage III	G2	Head	PD	No	Yes, liver	DFS nr OS 6 months	Yes
Ahmad et al., 2011 [8]	Case report	1	M	73	Epigastric pain, weight loss	No	T4N0M0 Stage III	nr	Body	Unspecified resection	No	No	DFS 6 months OS 6 months	No
Araki et al., 2011 [9]	Case report	1	M	68	Incidental finding	No	T2N0M0 Stage II	G3	Head	PD	No	No	DFS 52 months OS 52 months	No
Hirano et al., 2011 [10]	Case report	1	M	66	Jaundice, weight loss	No	T2N0M0 Stage II	G3	Head	PD	No, patient refused	Yes, unspecified	DFS 1 year OS 1 year	Yes
Yang et al., 2015 [11]	Retrospective study	6	nr	47.7 Mean	nr	Nr	Stage III 2 Stage IV 4	G3 6 100%	nr	2 PD 2 DPS 2 debulking biopsy	nr	nr	Mean DFS nr Mean OS 15.3	6 death
Imaoka et al., 2017 [12]	Case report	1	M	63	Incidental finding	No	T4N2M0 Stage III	G3	Head	PD	1 course of cisplatin and irinotecan, afterwards patient refusal	Yes, local, peritoneal carcinosis	DFS nr OS 6 months	Yes
Murata et al., 2017 [13]	Case report	1	M	66	Obstructive jaundice	No	T3N1M0 Stage III	G3	Head	PD	(2 courses of Tegafur gimercil oteracil (S-1) monotherapy	Yes, liver	DFS nr OS 12 months	Yes
Düzkoylü et al., 2018 [14]	Case report	1	M	72	nr	No	T3N0M0 Stage II	G1	Tail	DPS	Yes, unspecified	No	DFS 31 months OS 31 months	No
Niessen et al., 2020 [15]	Case series	8	7 M 1 F	Mean 70.5 (range 30–82)	nr	nr	Stage I 1 Stage III 6 Stage IV 1	G3 8100%	5 head 2 body 1 tail	5 PD 2 TP 1 DPS	7/8 4 gemcitabine 1 capecitabine 1 carboplatin + etoposide 1 cisplatin + etoposide	1 liver	DFS 6 pt 0 month 1 pt 88 months 1 pt 11 months Median OS 40 month	5 dead
Schiavo Lena et al., 2020 [16]	Case report	1	M	56	nr	Yes	T2N0M0 Stage II	G2	Head	PD	No	No	DFS 27 months OS 27 months	No
Varshney et al., 2020 [17]	Case report	1	M	81	Abdominal pain	No	nr	G1	Head	PD	4 cycles of with gemcitabine and cisplatin	No	DFS 12 months OS 12 months	No
Current Case	Case Report	1	F	59	Obstructive jaundice	No	T2N1M0 Stage III	G3	Head	PD	No, patient refused	No	DFS 12 months OS 12 months	No
	Total 28 pt		-M 18/22 (82.8%) -F 4/22 (18.2%)	Mean 61.7 (range 24–82)	-Abdominal pain 4/12 (33.3%) -Jaundice 4/12 (33.3%) -Incidental 3/12 (25%) -Weight loss 2/12 (16.6%) -Anaemia 2/12 (16.6%) -Nausea and vomiting 1/12 (8.3%)	Preoperative diagnosis 2/14 (14.2%)	-Stage I 2/26 (7.7%) -Stage II 5/26 (19.2%) -Stage III 14/26 (53.8) -Stage IV 5/26 (19.3%)	-G1 2/24 (8.3%) -G2 3/24–(12.5%) -G3 19/24 (79.2%)	-Head 14/22 (63.6%) -Body 4/22 (18.2%) -Tail 4/22 (18.2%)	-PD 16/28 (57.1%) -DPS 7/28 (25%) -TP 2/28 (7.1%) -Debulking procedure 2/28 (7.1%) -Unspecified resection 1/28 (3.7%)	-Neoadjuvant therapy 0% -Adjuvant therapy 11/20 (55%)	Recurrence 6/20 (30%)	Mean DFS 2.5 months median DFS 12 months (17 pt) range 0–216 months 1-year DFS 62.5% (10/16 pt) 2-year DFS 41.6% (5/12 pt) 5-year DFS 16.6% (2/12 pt) Mean OS 40.2 months median OS 12 months (26 pt) range 0–288 months 1-year OS 84% (21/25 pt) 3-year OS 27.7% (5/18 pt) 5-year OS 23.5% (4/17)	Dead 16/26 (61.5%)

Abbreviations: nr, not reported; PD, pancreaticoduodenectomy; DPS, distal splenopancreasectomy; DP, distal pancreatectomy; TP total pancreatectomy; OS; overall survival; DFS, disease-free survival.

## Data Availability

Not applicable.
